# m‐NLP Inference Models Using Simulation and Regression Techniques

**DOI:** 10.1029/2022JA030835

**Published:** 2023-01-27

**Authors:** Guangdong Liu, Sigvald Marholm, Anders J. Eklund, Lasse Clausen, Richard Marchand

**Affiliations:** ^1^ Department of Physics University of Alberta Edmonton AB Canada; ^2^ Department of Physics University of Oslo Oslo Norway; ^3^ Department of Computational Materials Processing Institute for Energy Technology Kjeller Norway; ^4^ Materials Physics Oslo SINTEF Industry Oslo Norway

**Keywords:** multi‐needle Langmuir probe, particle in cell simulation, multivariate regression, machine learning inference

## Abstract

Current inference techniques for processing multi‐needle Langmuir probe (m‐NLP) data are often based on adaptations of the Orbital Motion‐Limited (OML) theory which relies on several simplifying assumptions. Some of these assumptions, however, are typically not well satisfied in actual experimental conditions, thus leading to uncontrolled uncertainties in inferred plasma parameters. In order to remedy this difficulty, three‐dimensional kinetic particle in cell simulations are used to construct a synthetic data set, which is used to compare and assess different m‐NLP inference techniques. Using a synthetic data set, regression‐based models capable of inferring electron density and satellite potentials from 4‐tuples of currents collected with fixed‐bias needle probes similar to those on the NorSat‐1 satellite, are trained and validated. The regression techniques presented show promising results for plasma density inferences with RMS relative errors less than 20%, and satellite potential inferences with RMS errors less than 0.2 V for potentials ranging from −6 to −1 V. The new inference approaches presented are applied to NorSat‐1 data, and compared with existing state‐of‐the‐art inference techniques.

## Key Points

- 3‐D kinetic PIC simulations are used to simulate currents collected by multi‐needle Langmuir probe (m‐NLP) in order to create a synthetic solution library
- Models to infer physical parameters from m‐NLP measurements are constructed and assessed on the basis of synthetic and in situ data sets
- Promising new approaches are identified to analyze m‐NLP measurements based on simulation and in situ data

## 1 Introduction

Langmuir probes are widely used to characterize space plasma and laboratory plasma. A variety of Langmuir probe geometries are being used, such as spherical (Bhattarai & Mishra, 2017), cylindrical (Hoang, Clausen, et al., 2018), and planar probes (Johnson & Holmes, 1990; Lira et al., 2019; Sheridan, 2010). Probes can be operated in sweep mode (Lebreton et al., 2006), harmonic mode (Rudakov et al., 2001), or fixed biased mode (Jacobsen et al., 2010), for different types of missions and measurements. Despite operational differences, all Langmuir probes consist of conductors exposed to plasma to collect current as a function of bias voltage. A common approach to infer plasma parameters from Langmuir probes is to sweep the bias voltage and produce a current‐voltage characteristic, which can be analyzed using theories such as the Orbital Motion‐Limited (OML) (Mott‐Smith & Langmuir, 1926) theory, the Allen‐Boyd‐Reynolds (ABR) theory (Allen et al., 1957; Chen, 1965, 2003), and the Bernstein‐Rabinowitz‐Laframboise (BRL) theory (Bernstein & Rabinowitz, 1959; Laframboise, 1966) to obtain plasma parameters such as density, temperature, and satellite floating potential. The temporal and, on a satellite, the spatial resolution of Langmuir probe measurements are determined by the sweep time, which varies based on the mission's scientific need and available resources. Considering the orbital speed to be around 7,500 m/s for a satellite in low Earth orbit (LEO), the spatial resolution of sweep bias Langmuir probe can vary from tens of meters, to kilometers, depending on the sweep frequency. In order to study the formation of density irregularities that scale from meters to tens of kilometers at high and low latitudes, a sampling frequency of near 1 kHz is required (Hoang, Røed, et al., 2018; Jacobsen et al., 2010). A solution, proposed by Jacobsen is to use multiple fixed biased needle probes (m‐NLPs) to sample plasma simultaneously at different bias potentials in the electron saturation region (Jacobsen et al., 2010). This approach would eliminate the need for sweeping the bias voltage, and greatly increase the sampling rate of the instrument.

The first inference models for m‐NLPs relied on the OML approximation, from which the current *I*_*e*_ collected by a needle probe in the electron saturation region is written as:

(1) $$I_{e}=-n_{e}eA\frac{2}{\sqrt{\pi }}\sqrt{\frac{kT_{e}}{2\pi m_{e}}}{\left(1+\frac{e\left(V_{f}+V_{b}\right)}{kT_{e}}\right)}^{\beta },$$

where *n*_*e*_ is the electron density, *A* is the probe surface area, *e* is the elementary charge, *k* is Boltzmann's constant, *T*_*e*_ is the electron temperature, *V*_*f*_ is the satellite floating potential, *V*_*b*_ is the bias potential of the probe with respect to the satellite, and *β* is a parameter related to probe geometry, density, and temperature (Hoang, Røed, et al., 2018; Marholm & Marchand, 2020). Several assumptions were made in the derivation of this inference equation; such as the probe length must be much larger than the Debye length, and the plasma is non‐drifting. If these assumptions are valid, then *β* = 0.5, and as first suggested by Jacobsen, a set of m‐NLPs can be used to infer the electron density independently of the temperature (Jacobsen et al., 2010). For a satellite in near‐Earth orbit at altitudes ranging from 550 to 650 km, we can expect a Debye length of around 2–50 mm, and an orbital speed of around 7,500 m/s. A common length for m‐NLP instrument used on small satellites is ∼25 mm (Bekkeng et al., 2010; Hoang et al., 2019; Hoang, Clausen, et al., 2018), which is often comparable to, and sometimes smaller than the Debye length. In lower Earth orbit, ion thermal speeds are usually less than the orbital speed, while electron thermal speeds are usually higher than the orbital speed. Thus, the orbital speed is expected to mainly affect ion saturation region currents for Langmuir probes. However, electrons can only penetrate the ion rarefied wake region behind the probe as much as ambipolar diffusion permits (Barjatya et al., 2009). As a result, electron saturation currents are also influenced by an orbital speed. One consequence is that the *β* = 0.5 assumption does not hold in Equation 1, and a better approximation for the current is obtained with *β* values between 0.5 and 1. For example, in a hot filament‐generated plasma experiment, Sudit and Woods showed that *β* can reach 0.75 for a ratio between the probe length and the Deybe length in the range of 1–3. For larger Debye lengths, they also observed an expansion of the probe sheath from a cylindrical shape into a spherical shape (Sudit & Woods, 1994). Ergun and co‐workers showed that with a ram speed of 4,300 m/s in their simulations, the current collected by a 40.8 cm needle probe is better approximated with Equation 1 using a *β* value of 0.67 instead of 0.55 calculated in a stationary plasma (Ergun et al., 2021). In the ICI‐2 sounding rocket experiment, *β* calculated from three 25 mm m‐NLPs varied between 0.3 and 0.7 at altitudes ranging from 150 to 300 km (Hoang, Røed, et al., 2018). Simulation results by Marholm et al. showed that even a 50 mm probe at rest can be characterized by a *β* ∼ 0.8 (Marholm et al., 2019), in disagreement with the OML theory. In practice, needle probes are mounted on electrically isolated and equipotential guards in order to attenuate end effects on the side to which they are attached. The distribution of the current collected per unit length is nonetheless not uniform along the probe, as more current is collected near the end opposite to the guard. A study by Marholm and Marchand showed that for a cylindrical probe length that is 10 times the Debye length, *β* is approximately 0.72. For a probe length that is 30 times the Debye length, *β* is approximately 0.62, and with a guard, this number is reduced to 0.58 (Marholm & Marchand, 2020). Although this number approaches 0.5, 30 times the Debye length is a stringent requirement for OML to be valid, and it is hardly ever fulfilled in practice. Experimentally, Hoskinson and Hershkowitz showed that even with a probe length 50 times the Debye length, *β* is approximately 0.6, and the density inference based on an ideal *β* = 0.5 is 25% too high (Hoskinson & Hershkowitz, 2006). Barjatya estimated that even a 10% error in *β* (to 0.55) can result in a 30% or more relative error in the calculated density based on the *β* = 0.5 assumption (Barjatya & Merritt, 2018). In what follows, we find that densities estimated using Equation 1 assuming *β* = 0.5 are about three times larger than the known values used as input in our simulations, as illustrated in Section 2.4.1. This is consistent with findings in Barjatya and Merritt (2018) and Guthrie et al. (2021), considering *β* calculated in our simulation is in the range of 0.75–1. Another approach proposed to account for the fact that *β* is generally different from 0.5, consists of determining the *n*_*e*_, *V*_*b*_, *T*_*e*_, and *β*, as adjustable parameters in nonlinear fits of measured currents as a function of voltages. This led to remarkable agreement with density measured using a radio frequency impedance probe on the international space station (Barjatya et al., 2009, 2013; Debchoudhury et al., 2021). This method was originally applied to a probe operated in sweep voltage mode, but it can be straightforwardly adapted to fixed bias m‐NLP measurements (Barjatya & Merritt, 2018; Barjatya et al., 2009; Hoang, Røed, et al., 2018).

In the following, we assess different techniques to infer plasma densities, and satellite potentials from fixed bias needle probe measurements based on synthetic data obtained from kinetic simulations. We also present a new method to interpret m‐NLP measurements based on multivariate regression. Our kinetic simulation approach, the construction of a synthetic data set, and different models to infer plasma parameters are presented in Section 2. In Section 3, the various models are assessed using the synthetic data set. In Section 4, the same models are applied to NorSat‐1 data, to infer densities and satellite potentials from in situ measured currents. Section 5 summarizes our findings and presents some concluding remarks.

## 2 Methodology

In this section, we briefly describe our kinetic simulation approach, and how it is used to construct synthetic data sets used to train and validate inference models, using two regression techniques. We then describe the various models to infer density, and satellite potential from m‐NLP measurement.

### 2.1 Kinetic Simulations

**Figure 1:**
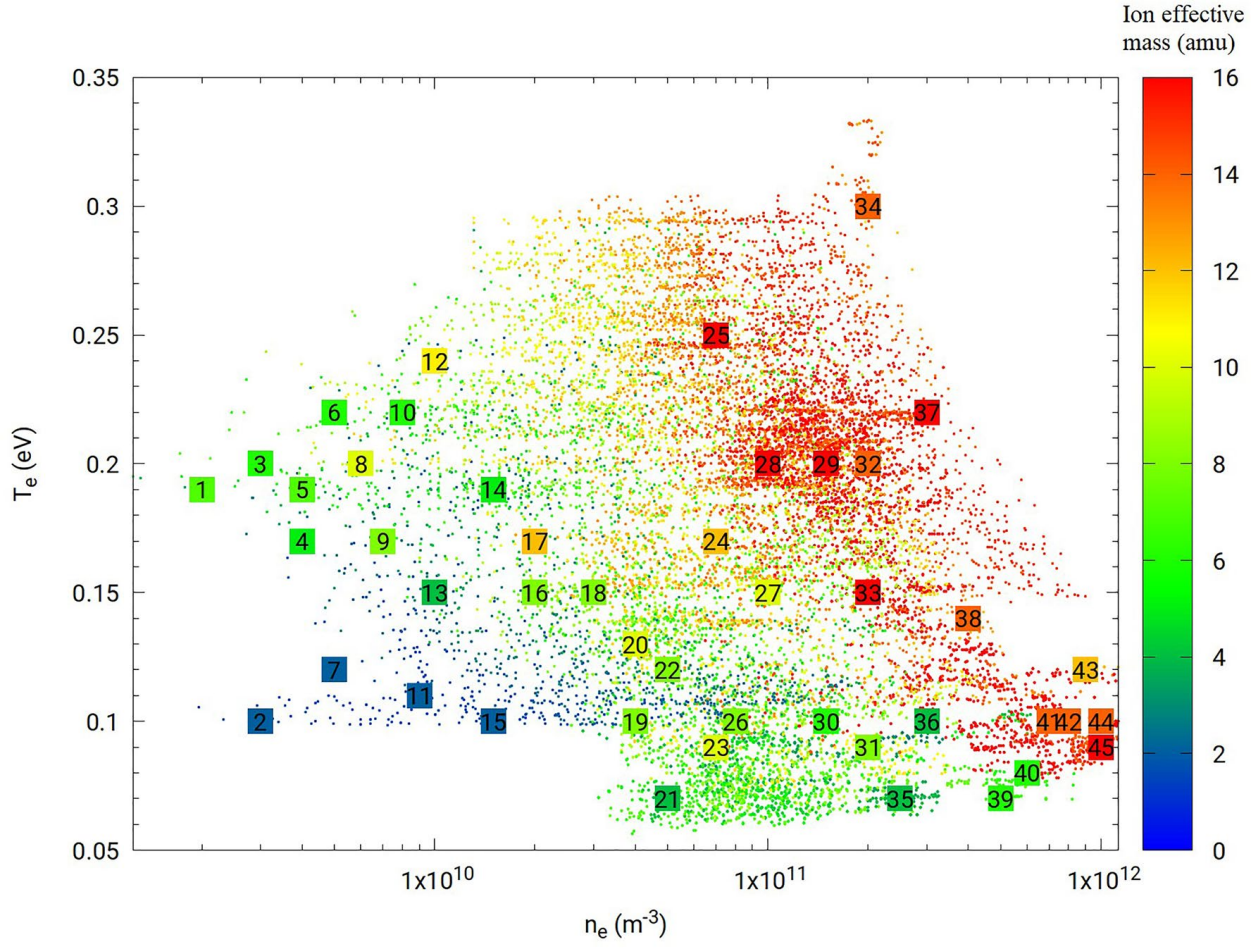
Scatter plot of plasma parameters obtained from the IRI model, corresponding to different latitudes, longitudes, altitudes, and times, as listed in Table 1. The *x* and *y* axes, and the color bar refer respectively, to the electron density, electron temperature, and the ion effective mass. Numbered squares identify the set of parameters used in the kinetic simulations.

**Figure 2:**
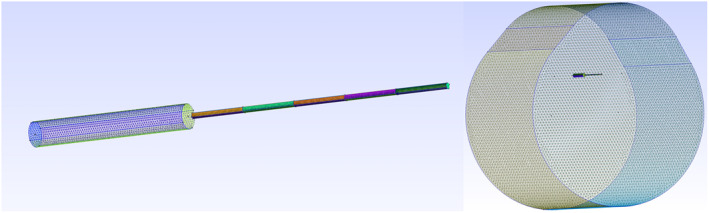
Illustration of an m‐NLP geometry (left), and the simulation domain (right). The needle probe has a length of 25 mm and a radius of 0.255 mm, with a guard of 15 mm in length and 1.1 mm in radius. The ram flow is from the top of the simulation domain and is assumed to be 7,500 m/s.

The space plasma parameters considered in our simulations are selected so as to be representative of conditions expected for a satellite in low Earth orbit at altitudes ranging between 550 and 650 km. This is done by sampling ionospheric plasma parameters using the International Reference Ionosphere (IRI) (Bilitza et al., 2014) model in a broad range of latitudes, longitudes, altitudes, and times as shown in Figure 1. The ranges considered for these parameters are summarized in Table 1. Forty‐five sets of plasma parameters approximately evenly distributed in this parameter space are selected as input in simulations, as shown in numbered squares in Figure 1. The three‐dimensional PIC code PTetra (Marchand, 2012; Marchand & Lira, 2017) is used to simulate probe currents in this study. Cross comparisons were made between PTetra simulation results and analytic results under conditions when those are valid, and with other independently developed simulation codes, and show excellent agreement (Deca et al., 2013; Marchand et al., 2014). In PTetra, space is discretized using unstructured adaptive tetrahedral meshes (Frey & George, 2007; Geuzaine & Remacle, 2009). Poisson's equation is solved at each time step using Saad's GMRES sparse matrix solver (Saad, 2003) in order to calculate the electric field in the system. Then, electron and ion trajectories are calculated kinetically using their physical charges and masses self consistently. The mesh for the m‐NLP and the simulation domain illustrated in Figure 2, is generated with GMSH (Geuzaine & Remacle, 2009). The needle probe used in the simulation has a length of 25 mm and a diameter of 0.51 mm, as those on the NorSat‐1. The needle probe is attached to a 15 mm long and 2.2 mm diameter guard which is biased to the same voltage as the probe. The outer boundary of the simulation domain is closer to the probe on the ram side, and farther on the wake side, as shown in Figure 2. The probes are assumed to be sufficiently far on the ram side, away from other satellite components, to be unaffected by their presence, and are identical except for their biases. A single probe is simulated at a time, and a synthetic solution library is then constructed by simulating the probe under different plasma conditions and voltages as described in Section 2.2. The simulations are made using two different domain sizes depending on the Debye length of the plasma. For plasma densities below 2 × 10^10^ m^−3^ corresponding to a Debye length of 1.9–7.2 cm, a larger domain is used. For plasma densities above 2 × 10^10^ m^−3^, corresponding to a Debye length of 0.2–2.2 cm, a smaller domain with finer resolution is used. The simulation size, the resolution, the number of tetrahedra, and the corresponding Debye length are summarized in Table 2. There is overlap between the two simulation domains for simulations with Debye lengths around 2 cm. No obvious difference was found in the simulated currents, indicating that simulation results from both domains are consistent in the transition range. Simulation results from both domains are included when training the regression models. All simulations start initially with 100 million ions and electrons, but these numbers vary through a simulation, due to particles being collected, leaving, or entering the domain. In the simulations, the probe is segmented into five segments of equal lengths as shown in Figure 2, making it possible to estimate a rough distribution of the current along its length. The current used to build regression models is a sum of the currents of the five different segments. The orbital speed of the satellite is assumed to be fixed at 7,500 m/s in the simulations, with a direction perpendicular to the probe. For the voltages considered, probes are expected to collect mainly electron currents. For simplicity, only two types of ions are considered in the simulation, *O* ^+^ and *H* ^+^ ions, and no magnetic field is accounted for, which is justified by the fact that the Larmor radius of the electron considered is much larger than the radius of the probe. NorSat‐1 satellite has a Sun‐synchronous orbit, thus moving approximately parallel to the magnetic field near the equator. As a result, in these regions $\vec{V}\times \vec{B}$ should be small at low and mid magnetic latitudes, and it is not accounted for in the simulations.

**Table 1:** Spatial and Temporal Parameters Used to Sample Ionospheric Plasma Conditions in IRI, and the Corresponding Ranges in Space Plasma Parameters

Environment and plasma conditions	Parameter range
Years	1998 2001 2004 2009
Dates	January 4 April 4 July 4 October 4
Hours	0–24 with increment of 8 hr
Latitude	−90° to +90° with increment of 5°
Longitude	0° to −360° with increment of 30°
Altitude	550–650 km with increment of 50 km
Ion temperature	0.07–0.16 eV
Electron temperature	0.09–0.25 eV
Effective ion mass	2–16 amu
Density	2 × 10^9^ to 1 × 10^12^ m^−3^

**Table 2:** Parameters Used in the two Simulation Domains

*D*_*ram*_ (cm)	*D*_*wake*_ (cm)	Probe resolution (µm)	Guard resolution (µm)	Boundary resolution	Tetrahedra	Debye length (cm)
3.5	7	51	220	2 mm	2.5 M	0.2–2.2
30	40	51	220	1 cm	1.7 M	1.9–7.2

*Note.* The first two columns give the distances between the probe to the outer boundary on the ram side (*D*_*ram*_), and the wake side (*D*_*wake*_) respectively, followed by the simulation resolutions at the probe, guard, and the outer boundary. The number of tetrahedra used in the simulations is in the order of millions. The corresponding range in Debye lengths is also listed.

### 2.2 Synthetic Solution Library

In order to assess the inference skill of a regression model, a cost function is defined with the following properties: (a) it is non‐negative, (b) it vanishes if model inferences agree exactly with known data in a data set, and (c) it increases as inferences deviate from actual data. The cost functions used in this work are: the root mean square error,

(2) $$RMS=\sqrt{\frac{1}{N_{\mathrm{data}}}\sum_{i=1}^{N_{\mathrm{data}}}{\left(Y_{{\mathrm{mod}}_{i}}-Y_{data_{i}}\right)}^{2}},$$

the root mean square relative error

(3) $$RMSr=\sqrt{\frac{1}{N_{\mathrm{data}}}\sum_{i=1}^{N_{\mathrm{data}}}\frac{{\left(Y_{{\mathrm{mod}}_{i}}-Y_{data_{i}}\right)}^{2}}{Y_{{\mathrm{mod}}_{i}}^{2}}},$$

the maximum absolute error

(4) $$MAE=\max\left\{\left|Y_{\text{mod}}-Y_{\mathrm{data}}\right|\right\},$$

and the maximum relative error

(5) $$MRE=\max\;\left\{\left|\frac{Y_{\text{mod}}-Y_{\mathrm{data}}}{Y_{\text{mod}}}\right|\right\},$$

where *Y*_*data*_ and *Y*_mod_ represent respectively known and inferred plasma parameters, and *N*_*data*_ is the total number of data points.

**Figure 3:**
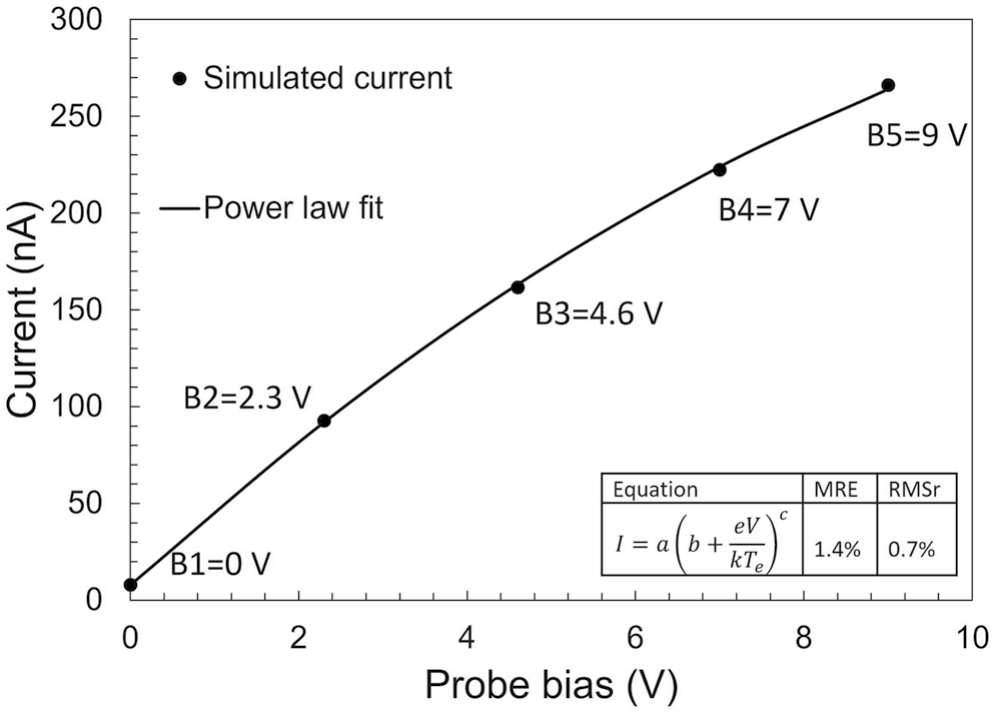
Comparison between calculated currents from PIC simulations, and fitted values using Equation 6, assuming a density of 2 × 10^10^ m^−3^, an effective mass of 8 amu, an electron and ion temperatures of 0.15 and 0.12 eV respectively, corresponding to point 16 in Figure 1. The fitting errors in the figure are calculated over all 45 sets of plasma conditions using Equations 3 and 5.

For each of the 45 sets of plasma conditions corresponding to squares in Figure 1, five simulations are made assuming five probe voltages (0, 2.3, 4.6, 7, 9 V) with respect to background plasma, and the simulated currents versus probe voltage are fitted analytically with:

(6) $$I=a{\left(b+\frac{eV}{kT_{e}}\right)}^{c},$$

where *a*, *b*, and *c* are adjustable fitting parameters. The MRE calculated for the 45 fits is 1.4%, and the RMSr is 0.7%, which shows excellent agreement with simulated collected currents. A comparison between fitted and computed currents is shown in Figure 3. The NorSat‐1 m‐NLP probes fixed biases *V*_*b*_ are +10, +9, +8, and +6 V, and the probe voltage with respect to background plasma is given by the sum of the spacecraft floating potential and the probe bias *V* = *V*_*f*_ + *V*_*b*_. In simulations, probe currents calculated for voltages with respect to background plasma in the range between 0 and 9 V are considered as shown in Figure 3. Considering the probe bias voltages *V*_*b*_ given above, probe currents can be determined, corresponding to arbitrary floating potentials between −1 and −6 V. A synthetic solution library is created for randomly distributed spacecraft floating potentials in the range between −1 and −6 V with corresponding currents obtained by interpolation using Equation 6 with the fitted *a*, *b*, and *c* computed for each of the 45 cases considered. The result is a synthetic solution library consisting of four currents collected by the four needle probes at the four different bias voltages, for 160 randomly distributed spacecraft potentials in the range between −1 and −6 V for each of the 45 sets of plasma parameters. In each entry of the data set, these four currents are followed by the electron density, the spacecraft potential the electron and ion temperatures, and the ion effective mass as listed in Table 3. The resulting solution library consisting of 45 × 160 = 7,200 entries is then used to construct a training set with 3,600 randomly selected nodes or entries, and a validation set with the remaining 3,600 nodes. The cost functions reported in what follows, used to assess the accuracy of inferences, are all calculated from the validation data set unless stated otherwise.

**Table 3:** Example Entries of the Synthetic Data Set, With Currents *I*_1_, *I*_2_, *I*_3_, and *I*_4_ Calculated Using Equation 6, and *V*_*b*_ Set to 10, 9, 8, and 6 V, Respectively

*I*_1_(*nA*)	*I*_2_(*nA*)	*I*_3_(*nA*)	*I*_4_(*nA*)	*V*_*f*_(*V*)	*n*_*e*_(m^−3^)	*T*_*e*_ (eV)	*T*_*i*_ (eV)	m_eff_ (amu)
233	208	183	129	−2.50	2 × 10^10^	0.15	0.12	8
596	533	467	323	−2.93	5 × 10^10^	0.07	0.07	4

*Note.* The floating potential *V*_*f*_ is selected randomly in the range of −1 to −6 V, and the probe voltages with respect to background plasma are given by *V* = *V*_*b*_ + *V*_*f*_. The coefficients, *a*, *b*, and *c* are obtained from a nonlinear fit of the simulated currents using Equation 6. The first and second entries correspond respectively to points 16 and 21 in Figure 1.

### 2.3 Multivariate Regression

In a complex system where the relation between independent variables and dependent variables cannot readily be cast analytically, multivariate regressions based on machine learning techniques are powerful alternatives to construct approximate inference models. In this approach, the model must be capable of capturing the complex relationship between dependent and independent variables. Once the model is trained using the training set, it can then be used to make inferences for cases not included in the training data set. In this work, two multivariate regression approaches are used to infer plasma parameters: the Radial Basis Function and Feedforward Neural Networks. The models are trained by minimizing their cost function on the training data set, and then applied to the validation data set to calculate the validation cost function without further optimization. The use of a validation set is to avoid “overfitting” because there are certain limitations on the refinement of a model on a training set, such that further improvement of model inference skill in the training set will worsen the model inference skill in the validation set. A good model is one with the right level of training so as to provide the best inference skill in the validation set.

#### 2.3.1 Radial Basis Function

Radial basis function (RBF) multivariate regression is a simple and robust tool used in many previous studies to infer space plasma parameters using a variety of instruments with promising results (Chalaturnyk & Marchand, 2019; Guthrie et al., 2021; Liu & Marchand, 2021; Olowookere & Marchand, 2021). A general expression for RBF regression for a set of independent *n*‐tuples $\bar{X}$ and corresponding dependent variable *Y* is given by:

(7) $$Y=\sum_{i=1}^{N}a_{i}G\left(\left|\bar{X}-\bar{X_{i}}\right|\right).$$

In general, the dependent variable *Y* can also be a tuple, but for simplicity, and without loss of generality, we limit our attention to scalar dependent variables. In Equation 7, the ${\bar{X}}_{i}$ represents the *N* centers, *G* is the interpolating function, and the *a*_*i*_ are fitting collocation coefficients which can be determined by requiring collocation at the centers; that is, by solving the system of linear equations

(8) $$\sum_{i=1}^{N}a_{i}G\left(\left|{\bar{X}}_{k}-{\bar{X}}_{i}\right|\right)=Y_{k}$$

for *k* = 1, …, *N*. Here, the dependent variable *Y* corresponds to the physical parameter to be inferred, and the independent variable $\bar{X}$ is a 4‐tuple corresponding to the currents or the normalized currents from the m‐NLPs depending on which physical parameters are being inferred. There are different ways to distribute the centers in RBF regression. One straightforward approach is to select centers from the training data set, and evaluate the cost function over the entire training data set for all possible combinations of centers, then select the model which yields the optimal cost function. For this approach, the number of combinations required for N data points and *N* centers is given by

(9) $$\left(\begin{array}{c} N\\ N \end{array}\right)=\frac{N!}{N!\left(N-N\right)!}.$$

This, of course, can be prohibitively large and time‐consuming for a large training data set or using a large number of centers. An alternative strategy is to successively train models with randomly selected small subsets of the entire training data set using the straightforward approach, while calculating the cost function on the full training set, and then carrying the optimal centers from one iteration to the next. This “center‐evolving strategy” is very efficient in finding near‐optimal centers for large training data sets and has proven to be as accurate as the straightforward extensive approach (Liu & Marchand, 2022). The RBF models here follow this procedure. Different G functions and cost functions are tested, and only the models that yield optimal results are reported in this paper.

#### 2.3.2 Feedforward Neural Network

**Figure 4:**
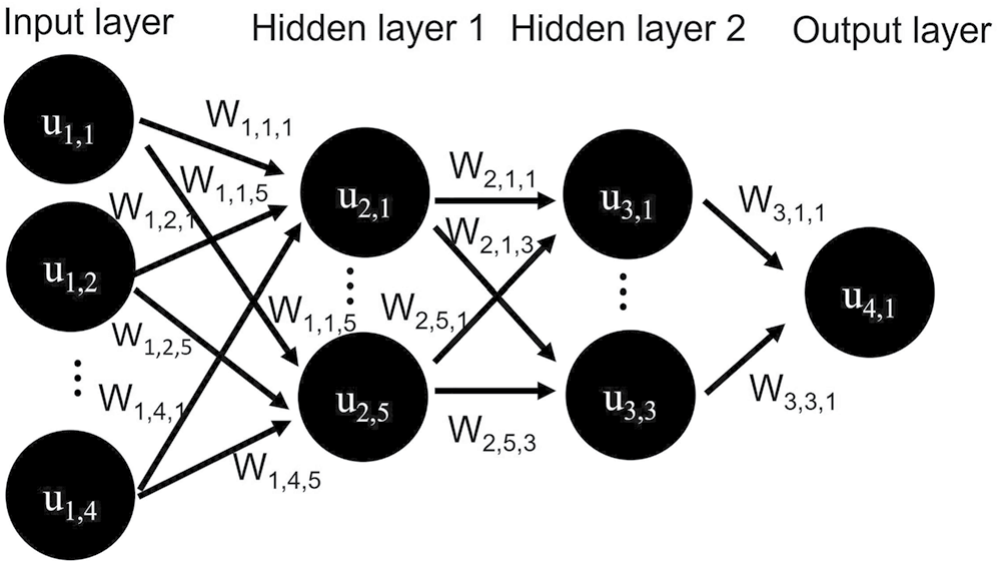
Schematic of a feedforward neural network.

The second multivariate regression approach is a Feedforward neural network as illustrated in Figure 4. This consists of an input layer, hidden layers, and an output layer. Each node *j* in a given layer *i* in the network is assigned a value *u*_*i*,*j*_, and the node in the next layer *i* + 1 are “fed” from numerical values from the nodes in the previous layer according to

(10) $$u_{i+1,k}=f\left(\sum_{j=1}^{n_{i}}w_{i,j,k}u_{i,j}+b_{i,k}\right),$$

where *w*_*i*,*j*,*k*_ are weight factors, *b*_*i*,*j*_ are bias terms, and *f* is a nonlinear activation function (Goodfellow et al., 2016). In this work, the input layer neurons contain the four‐needle probe currents or normalized currents depending on the physical parameter to be inferred, whereas the output layer contains one physical parameter. The number of hidden layers and the number of neurons in the hidden layers are adjusted to fit the specific problem, and attain good inference skills. The Feedforward neural network is built using TensorFlow (Abadi et al., 2016) with Adam optimizer (Kingma & Ba, 2015), and using the ReLU activation function defined as *f*(*x*) = max(0, *x*). The input variables are normalized using the preprocessing. normalization TensorFlow built‐in function which normalizes the data to have a zero mean and unit variance. The structure of the network will be described later when presenting model inferences.

### 2.4 Space Plasma and Satellite Parameter Inference Models

The next step is to construct models that map the measured currents to the corresponding plasma and satellite conditions in the solution library. Various models used to infer plasma densities and satellite potentials are described in this section.

#### 2.4.1 Density Inference

The density can be inferred directly from the above two multivariate regression models using the currents collected by the four probes as inputs. The density can also be inferred using Equation 1 which can be rewritten as

(11) $$\frac{n_{e}}{T_{e}^{\beta -\frac{1}{2}}}=\sqrt{\frac{\pi^{2}m_{e}}{2A^{2}e^{3}}}{\left(\frac{I_{1}^{\frac{1}{\beta }}-I_{2}^{\frac{1}{\beta }}}{V_{1}-V_{2}}\right)}^{\beta }.$$

**Figure 5:**
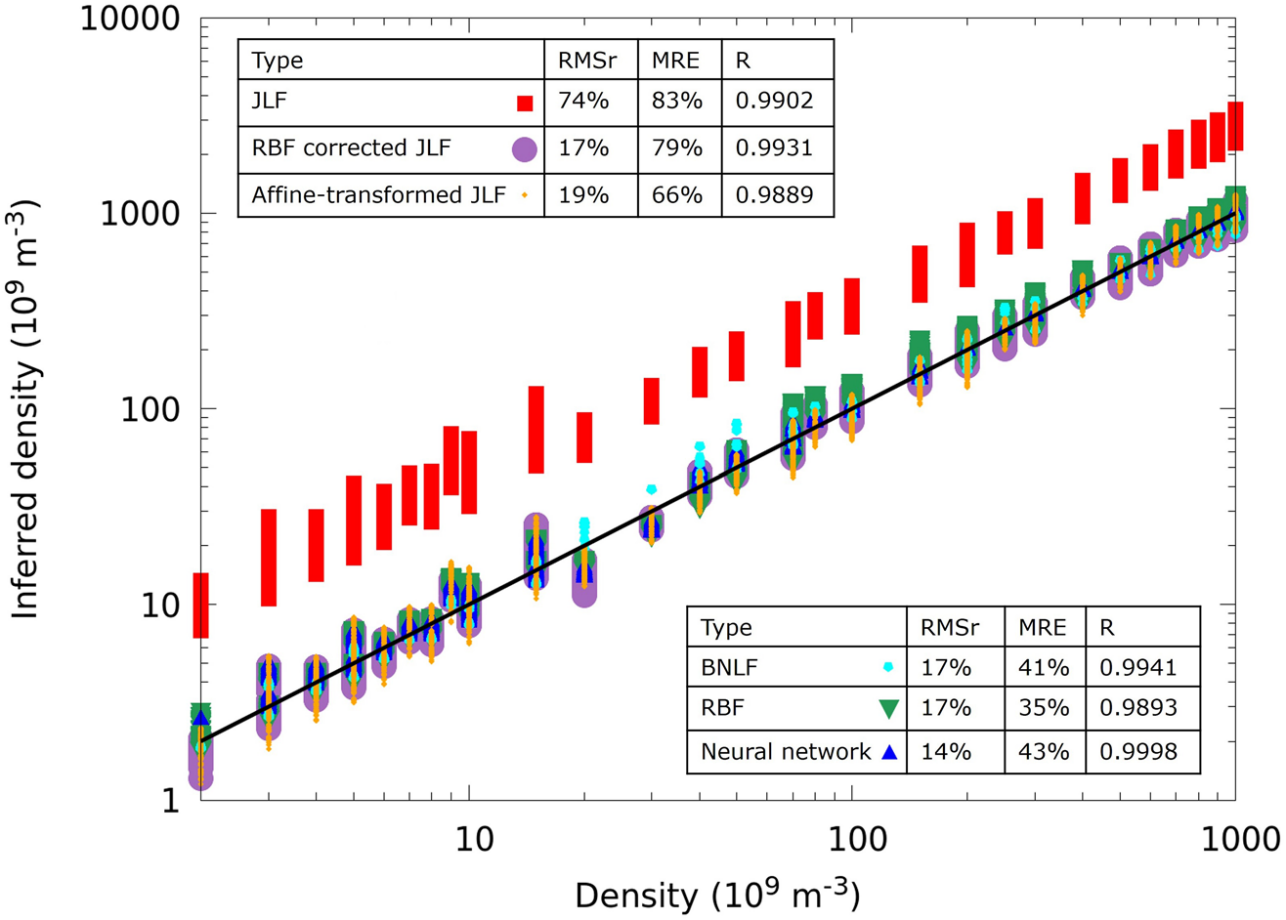
Correlation plot for the density inferences made with different techniques applied to our synthetic validation set. The Pearson correlation coefficient *R* is calculated using the inferred densities and the density used in the simulation. The black line represents the idealized perfect correlation.

In this equation, subscripts 1 and 2 indicate different probes. A special case of this equation was first proposed by Jacobsen, assuming an infinitely long probe and a stationary plasma, for which *β* = 0.5, resulting in

(12) $$n_{e}=\sqrt{\frac{\pi^{2}m_{e}}{2A^{2}e^{3}}}\sqrt{\frac{I_{1}^{2}-I_{2}^{2}}{V_{1}-V_{2}}},$$

which gives an expression for the electron density, independently of the temperature (Jacobsen et al., 2010). With currents from more than two probes, the density can be calculated from the slope of the current squared as a function of the bias voltage from a linear least‐square fit of all probes (Jacobsen et al., 2010). This will be referred as the “Jacobsen linear fit” (JLF) approach. It is now well known, however, that for finite length probes, with lengths not much larger than the Debye length, *β* typically ranges between 0.6 and 1. This is the case in particular for the needle probes on NorSat‐1 with ratios between probe lengths to Debye length ranging from 0.5 to 12.5. As a consequence, when this method is applied to the solution library, the inferred density is typically three times larger than the density used in the simulation as shown with red boxes in Figure 5. Analytic inferences can be improved by adopting a boosting strategy. With this approach, the less accurate analytic model is used as a first approximation, which is then corrected by applying a more advanced regression technique.

Two boosting strategies are used in this study, consisting of (a) an affine transformation, and (b) RBF. Considering that the Pearson correlation coefficient *R* is invariant under an affine transformation, it follows that the offset between two data sets, with a high value of *R*, can be significantly reduced with a simple affine transformation. To be specific, in this case, the density is first approximated using the JLF approach, and an affine transformation is applied to the natural log of the density as in:

(13) $$\ln\left(n_{e}^{\text{affine}}\right)=a\ln\left(n_{e}^{\text{JLF}}\right)+b.$$

where *a* and *b* are determined with a simple least square fit to the known log of the densities in the data set. In the second approach, RBF is used to model the discrepancy between the JLF approximated density and the known densities, and the modeled discrepancy is used to correct the first JLF estimate.

The nonlinear least squares fit proposed by Barjatya (BNLF) is also used to infer the density and the satellite potential. In their paper, Barjatya et al. (2009) apply this method to a full characteristic, covering the ion saturation, the electron retardation, and electron saturation regions. This enabled them to infer all four parameters in Equation 1, namely, *n*_*e*_, *T*_*e*_, *V*_*f*_, and *β*. In our analysis, inferences are made from only four currents from four probes at fixed bias voltages, all in the electron saturation region. As shown by Barjatya and Merritt (Barjatya & Merritt, 2018), however, it is difficult to infer the temperature using this approach, owing to the weak dependence of collected currents on the electron temperature (see Equation 11). A solution, proposed in Barjatya and Merritt (2018) and Hoang, Røed, et al. (2018), then consists of estimating the electron temperature from other measurements, or from the IRI model, and performing the fit for the remaining three parameters. This simplification is justified by the fact that, following this procedure, a 50% error in the temperature, still produces acceptable results for the other parameters (Barjatya & Merritt, 2018). When applying the BNLF method in the comparisons below, we assume a fixed electron temperature (∼3000 K) in our fit, and then use the 4‐tuples of currents using *V*_*f*_, *n*_*e*_, and *β* values as fitting parameters. The choice of electron temperature also affects the performance of the model. This choice of temperature (∼3000 K) is justified by the fact that it gives the best estimates of density and potential when BNLF is applied to our synthetic data set. In their original approach, Barjatya et al. (2009) assumed half of the probe on the ram side collects electrons due to the wake effect. With this assumption, they found that the inferred electron densities were more consistent with their inferred ion densities. In the simulation approach, the wake and its effect on electron collection are accounted for self‐consistently, and excellent inferences can be made without this assumption.

#### 2.4.2 Analytic Estimate of *V*_*f*_

The satellite potential can be inferred directly from the currents using RBF regression. In this approach, the four currents are normalized by dividing every current by their sum, in order to remove the strong density dependence on the currents. A neural network does not produce satisfactory in this case, and it is not used to infer the satellite potential. The floating potential of the spacecraft can also be inferred using the OML equation, by rewriting Equation 1 as:

(14) $$V_{f}\approx V_{f}+\frac{kT_{e}}{e}=\frac{V_{2}I_{1}^{\frac{1}{\beta }}-V_{1}I_{2}^{\frac{1}{\beta }}}{I_{2}^{\frac{1}{\beta }}-I_{1}^{\frac{1}{\beta }}}=\frac{V_{3}I_{2}^{\frac{1}{\beta }}-V_{2}I_{3}^{\frac{1}{\beta }}}{I_{3}^{\frac{1}{\beta }}-I_{2}^{\frac{1}{\beta }}}.$$

In this equation, the subscripts 1, 2, and 3 refer to different probes, thus there must be at least three probes in order to solve for *β*. The bias voltages of the probes and their corresponding collected currents are known from measurements, thus *β* can be solved using a standard root finder. Given *β*, Equation 14 then provides a value for $V_{f}+\frac{kT_{e}}{e}$. In this expression, $\frac{kT_{e}}{e}$ is the electron temperature in electron‐volt, which in the lower ionosphere at mid latitudes, is of order 0.3 eV or less. Thus, considering that $\frac{kT_{e}}{e}$ is generally much smaller than satellite potentials relative to the background plasma, any of the two terms on the right side of Equation 14 provides a first approximation of *V*_*f*_ (Guthrie et al., 2021). This will be referred to as the “adapted OML” approach.

## 3 Assessment With Synthetic Data

In this section, we assess our models using synthetic data, which allows us to check the accuracy, and quantify uncertainties in our inferences. A consistency check strategy is also introduced to further assess the applicability of our models.

### 3.1 Density and Satellite Potential Inference

Direct RBF regression is applied to infer the density using the four currents as input variables. When constructing an RBF model with *G*(*x*) = |*x*|, minimizing MRE, and using 6 centers, the RMSr and MRE calculated on the validation data set are 17% and 35%, respectively. A test is made to infer the density using RBF with 35 randomly selected entries from the 45 plasma conditions in the solution library. With 30 voltages, and the same *G* function, cost function, and number of centers, the calculated MRE is also 35%. This is an indication that 45 sets of plasma conditions and 160 voltages should be sufficient in terms of sampling size to construct regression models. Using a neural network with 4 nodes in the input layer, 14 nodes and 12 nodes in two hidden layers, and 1 node in the output layer, results in a 14% RMSr and 43% MRE for the inferred densities. This is calculated using TensorFlow with ADAM optimizer with a learning rate of 0.005 and an RMSr as the cost function. The input layer is normalized to have a zero mean and unit variance, while the output layer is normalized by dividing by the largest density. The densities calculated using the synthetic solution library, as well as the cost function are shown in Figure 5.

When using an affine transformation to boost the JLF method, the coefficients a and b in Equation 13 are obtained from a least squares fit of the log of these densities, to those in the training data set. The fitting coefficients in this case, *a* = 1.13261 and *b* = −4.82735, are then used to perform an affine transformation on the validation data set, leading to a significant improvement in RMSr from 74% to 19%, and in MRE from 83% to 66% compared to densities inferred from the JLF approach, as shown in Figure 5. When boosting JLF density with RBF, the 4‐tuple of currents is used as input variable $\bar{X}$. Minimizing the MRE using *G*(*x*) = |*x*|, and 5 centers, the RBF corrected JLF density yields an RMSr of 17% and an MRE of 79%. The cost functions of the two boosting methods are comparable, but an obvious advantage of using an affine transformation is its simplicity.

The Python 3 LMFIT package is used to do the nonlinear fit for the BNLF approaches as in Debchoudhury et al. (2021). In the fits, the initial values for the density, the potential, and the *β* value are 8 × 10^10^ m^−3^, −3 V and 0.85, and the lower and upper bounds are 1 × 10^8^ to 1 × 10^12^ m^−3^, −6 to −1 V, and 0.49 to 0.99, respectively. The tolerance of the fit is set to ftol = 1e−90, and the maximum number of function evaluations before termination is set to max_nfev = 100,000, to ensure a sufficient number of evaluations before termination. The potential lower bound of −6 V is needed to ensure that the values under exponent in Equation 1 are positive. We obtain 3,600 fits for each of the 3,600 entries of four currents in our validation data set. The overall RMSr calculated using Equation 3 for the 3,600 × 4 currents is 1.4%, and the MRE is 3.8%. The resulting density inferences have an RMSr of 17% and an MRE of 41%, which is better than the densities inferred from the affine‐transformed JLF approach, but less accurate than those from the multivariate regression models. The *β* values calculated are in the range of 0.75–1. Using LMFIT and multiprocessing packages, and 10 parallel processors in the Pool, 3,600 fits can be done in 0.96 s using an AMD 5800x processor. In comparison, linear fits of the currents square, followed by an affine transformation of the log of the inferred density can be done using fixed formulas (7,400 sets can be fitted in one second using an AMD 5800x without parallelization), and thus are considerably faster than a nonlinear fit. Regression methods such as RBF or neural networks are also numerically very efficient, considering they involve simple arithmetic expressions with pre‐calculated coefficients. Compared to the other density models considered, straightforward RBF yields the smallest MRE, thus it is the preferred model to infer density in this work. However, the affine‐transformed JLF method enables density inferences with accuracy comparable to those of more complex approaches. This simple and practical technique should therefore be of interest in routine data analysis.

**Figure 6:**
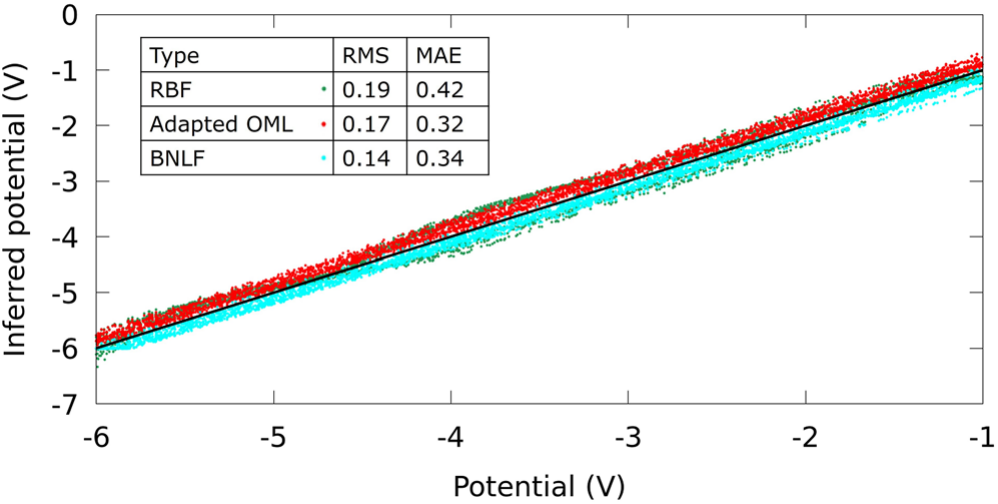
Correlation plot obtained for satellite potential inferred with RBF and OML techniques.

When the adapted OML approach is used to infer satellite potentials, an MAE of 0.3 V is calculated using currents collected with probe biases of 10, 9, and 8 V probes. Referring to Equation 14, the error of 0.3 V is likely due in part to the maximum electron temperature of 0.3 eV considered in the simulations. The *β* values calculated in the synthetic solution library are in the range of 0.75–1. RBF regression is also used to infer satellite potentials. In this case, using *G*(*x*) = |*x*|, 5 centers, and minimizing the MAE, the calculated MAE on the validation data set is 0.42 V, and the RMS is 0.19 V. The inferred satellite potential from the BNLF approach has an RMS of 0.14 V, and an MAE of 0.34 V, which proves this method to be the most accurate compared to the other methods considered. A correlation plot for potentials inferred using the RBF, adapted OML, and BNLF approaches is shown in Figure 6. All methods show good agreement with values from the synthetic solution library.

### 3.2 Consistency Check

**Figure 7:**
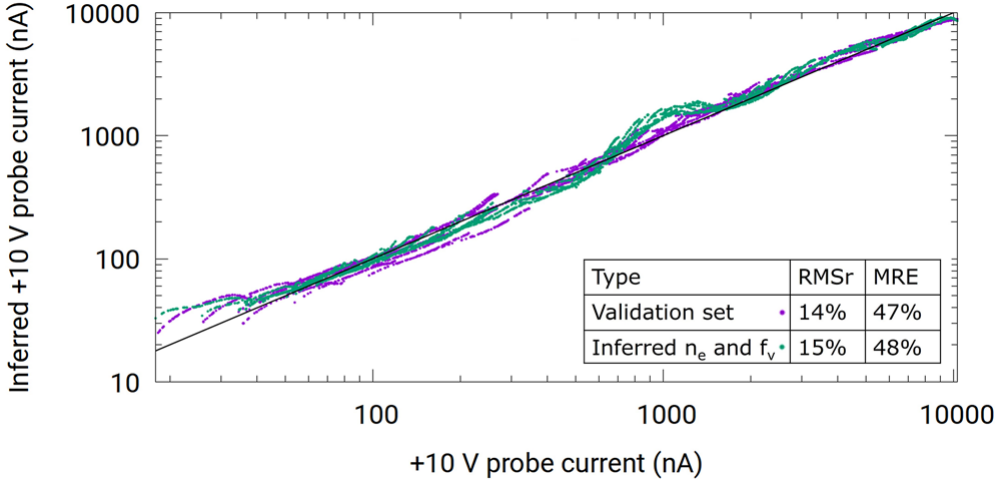
Correlation plot of inferred +10 V probe current against +10 V probe current from the synthetic data set. The calculated +10 probe currents in the purple curve are obtained using the validation data set, while the green curve is calculated using inferred densities and floating potentials from RBF regression.

In order to further assess the applicability of our inference approaches, we perform the following consistency check. First, RBF models *M*1(*n*_*e*_) and *M*1(*V*_*f*_) are constructed to infer the density and satellite potential using 4‐tuple currents from our synthetic data set. A second model (*M*2) is constructed to infer collected currents from densities and floating potentials in our synthetic data set. Since we are not able to infer temperatures from the currents, the temperature is not included in *M*2. Consistency is then assessed in two steps, by (a) using currents from synthetic data and models *M*1(*n*_*e*_) and *M*1(*V*_*f*_) to infer densities and floating potentials, and (b) applying models *M*2 to these inferred values, to infer back collected currents. RBF density and floating potential inferences are used in *M*1(*n*_*e*_), and *M*1(*V*_*f*_) as described in Section 2.4. RBF is also used in *M*2 with $G\left(x\right)=\sqrt{1+x^{2.5}}$, and minimizing RMSr with five centers. With perfect inference models, the results for these back‐inferred currents, should agree exactly with the starting currents from synthetic data. Variances between back‐inferred and simulated currents in the synthetic data are presented as indicative of the level of confidence in our regression techniques. The correlation plot in Figure 7, shows back‐inferred currents (green) calculated for a probe with 10 V bias against known currents from synthetic data. For comparison, the figure also shows the correlation between directly inferred currents (purple) when model *M*_2_ is applied to densities and floating potentials in the synthetic data set. Both back‐inferred and directly inferred currents are in excellent agreement with known currents from synthetic data, with comparable metric skills of ≃15% and ≃48% for the RMSr and the MRE, respectively. Considering that errors are compounded between the first and second models for the back‐inferred currents, the nearly identical metric skills in Figure 7 is seen as confirmation of the validity of our regression models.

## 4 Application to NorSat‐1 Data

In this section, we apply our density and potential inference models constructed with synthetic data, to in situ measurements made with the m‐NLP on the NorSat‐1 satellite. The NorSat‐1 currents were obtained from a University of Oslo data portal (Hoang, Clausen, et al., 2018). The epoch considered corresponds to one and a half orbit of the satellite starting at approximately 10:00 UTC on 4 January 2020. We start with a comparison of simulated and measured currents to verify that our simulated currents are in the same range as those of measured in situ currents. Inferences made with RBF, neural network, BNLF, adapted OML, and the two corrected JLF approaches constructed in Section 2.4, are also presented.

### 4.1 Measured In Situ, and Simulated Currents

**Figure 8:**
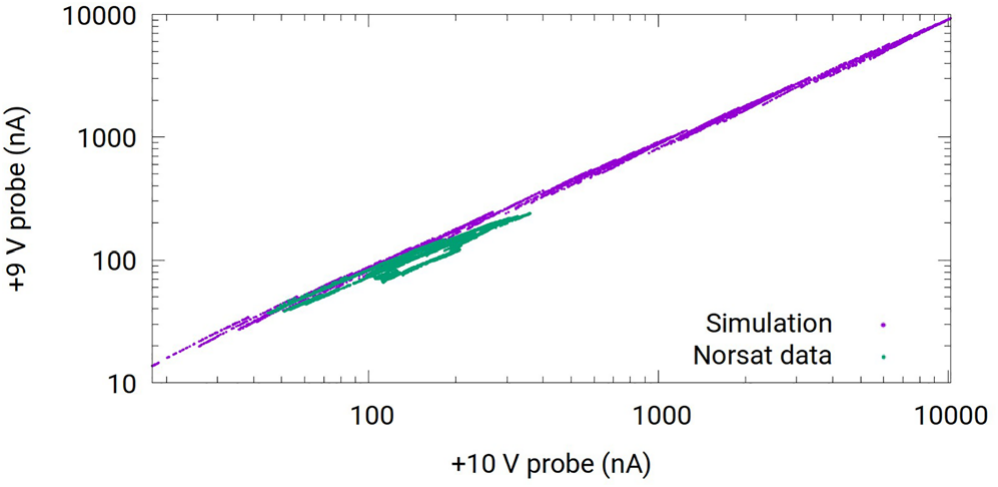
Correlation plot between currents collected by the +9 V and the +10 V probes for both NorSat‐1, and synthetic data.

The relevance of the space plasma parameter range considered in the simulations, to NorSat‐1, is assessed in Figure 8, by plotting currents collected by the +9 V probe against that collected by the +10 V, from both synthetic data, and in situ measurements. The close overlap, and the fact that the range of in situ measurements is within the range of simulated currents, indicate that the physical parameters selected in the simulations, are indeed representative of conditions encountered along the NorSat‐1 orbit.

The current measurement resolution for the NorSat‐1 m‐NLP probes is approximately 1 nA (Hoang, Clausen, et al., 2018). The noise level from the environment, however, is estimated to be of order 10 nA. In what follows, darker colors are used to represent inferences made using currents above 10 nA, and lighter colors are used to represent inferences using currents between 1 and 10 nA. This is done by filtering out all data that contain a current that is below 10 nA or 1 nA in any of the four probes. A word of caution is in order, however, for inferences made from these lower currents, as a conservative estimate of the threshold for sufficient signal‐to‐noise ratios, is approximately 10 nA. This lower bound current is supported by a consistency check made with models 1 and 2 described in Section 3.2, and presented below in Section 4.3.

### 4.2 Density and Satellite Potential Inference

**Figure 9:**
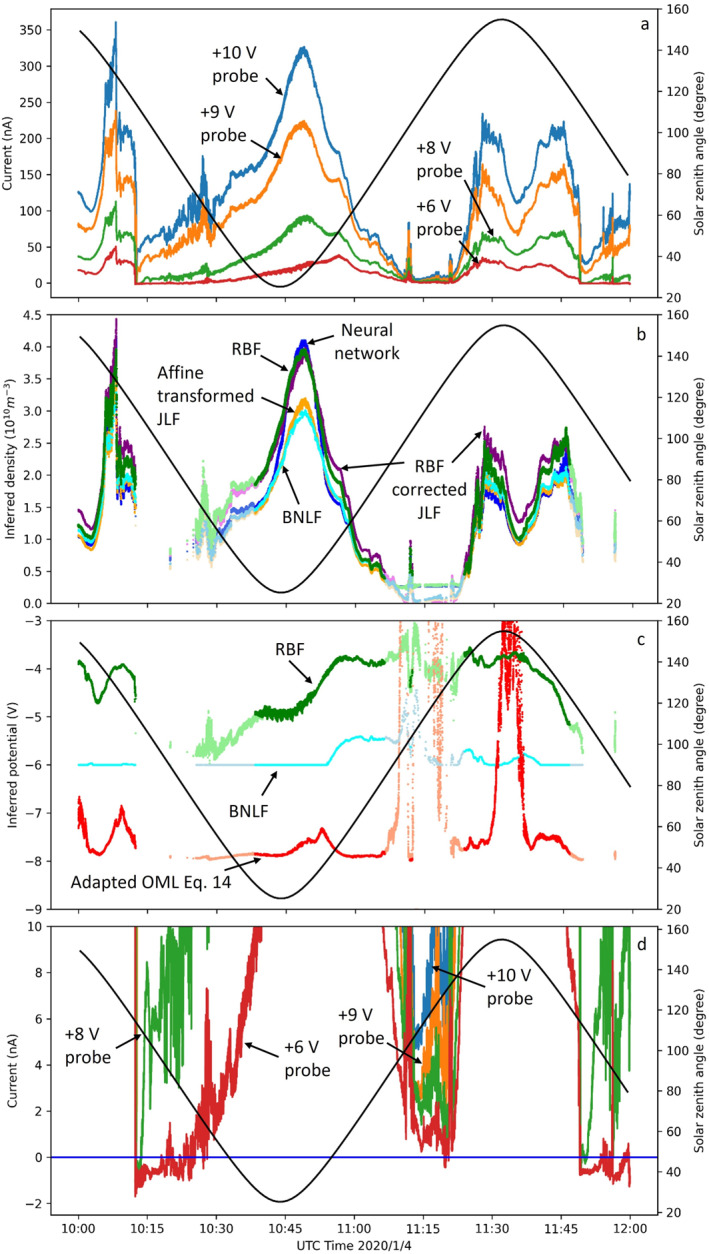
Illustrations of NorSat‐1 collected currents considered in this study in panel (a), inferred densities in panel (b), inferred potentials in panel (c), and the NorSat‐1 current near 0 A in panel (d) The solar zenith angle is also plotted against the secondary axis. Curves in darker colors are from model inferences using data above 10 nA, whereas those in lighter colors show inferences using data with currents between 1 and 10 nA.

Our models, trained with synthetic data as described in Section 2.4, are now applied to infer plasma densities and satellite potentials from in situ measured currents, for the time period considered. The results obtained with the different models presented in Section 2.4 are shown in Figure 9 for the inferred densities, satellite potentials, and measured currents collected by the four probes. The position of the satellite relative to the Earth and the Sun given by the solar zenith angle is also plotted in the figure. For example, a small solar zenith angle means that the satellite is near the equator on the dayside.

Applying the BNLF method with only four probes at fixed bias voltages, all in the electron saturation region, is more challenging than applying the technique to a probe operated in sweep mode, covering the ion saturation, the electron retardation, and the electron saturation regions. The reason is that in sweep mode, characteristics contain much more information than in fixed bias mode, with only four probes. In practice, inferences made from sweep mode characteristics are less sensitive to random errors in the currents, which, owing to their larger numbers, tend to cancel. With only four currents, however, noise is less likely to cancel, and inferences will be more sensitive to errors or noises in measured currents. For example, the +8 V NorSat‐1 probe currents are often slightly lower than expected for a downward concavity in *I* as a function of *V*_*b*_, and tend to produce an upward concavity with *β* larger than 1. Thus in most cases, the fitted *β* value reaches the maximum allowed value of 1. The resulting inferred densities and satellite potentials are shown in Figure 9. For the reasons mentioned above, it is clear that no satellite potential below the fitting lower bound of −6 V can appear in the plot.

The densities shown in Figure 9b are obtained using the five density inference methods mentioned in Section 2.4.1. At 10:45, the neural network density, the RBF corrected JLF density, and the RBF density overlap nicely, while the affine transformed JLF density and the BNLF density are smaller than other inferred densities, particularly near the density maxima. The density inferences nonetheless qualitatively agree with each other. Note that at around 11:15, the inferred densities fall below 2 × 10^9^ m^−3^, which is outside the range of the synthetic solution library. As a result, the regression models such as RBF and neural networks do not produce the right densities in these ranges. A note of caution should also be placed for other models at these lighter color regions since the signal‐to‐noise ratio is low for data with currents below 10 nA.

Using the +10, +9, and +8 NorSat‐1 probe currents and Equation 14, the inferred satellite floating potential is about −8 V for most of the data range considered in this study as shown in Figure 9c. This is in stark contradiction with observations in Figure 9d, which shows that the +6 V biased probe collects net positive electrons during most of the period considered. Also, there are periods between 10:15 to 10:30, and after 11:45 when the +6 V probe collects ion current(negative), indicating drops in the satellite potential below −6 V. The poor performance of Equation 14 to infer the satellite potential here, results from the fact that Equation 14 yields erratic values of *β* ranging from 0.3 to 1.2. Attempts have also been made to approximate the satellite potential with Equation 14 using a fixed value of 0.58 and 0.78 for *β*, also resulting in satellite potentials in the −8 V range, and no improvement was found. This failure to produce acceptable values of the satellite potential clearly shows that the generalized OML expression in Equation 14 does not provide a sufficiently accurate approximation for the currents collected by the NorSat‐1 probes.

The floating potentials inferred from the BNLF model are systematically lower than those from RBF and are often bounded by the fitting lower limit of −6 V. This is likely caused by the fact that the +8 V probe current is often lower than expected for a downward concavity in *I* as a function of *V*_*b*_. The RBF inferred floating potential shown in Figure 9, is within −4 and −6 V, which is consistent with the observation that the +6 V probe collects electrons during most of the time period considered. This potential is generally lower than the potential established by the spacecraft on its own, likely due to the large number of electrons collected by the positively biased solar panels (Ivarsen et al., 2019). Interestingly, the inferred satellite potential using currents between 1 and 10 nA (light color) is seen to join smoothly with the darker color inferences, and to decrease below −6 V around 10:25, which is consistent with the observation that during that time the +6 V probe no longer collects electron current. The currents collected by the probes are determined mostly by the density and the satellite potential, and to a lesser extent, by the electron temperature. In Figure 9, the density and floating potential are seen to peak at around 10:45 and 11:00 respectively. The currents from the +8, +9, and +10 V probes (green, orange, and blue) peak around 10:45, coinciding with the peak in the plasma density at this time. Then, as time goes forward to 11:00, the currents of the three probes decrease, also coinciding with a decrease in plasma density. However, the +6 V probe (red) current is increasing during these times, possibly due to an increase in floating potential. This increase is captured in the RBF and BNLF inferred potential, but not in the one derived from adapted OML. Another observation is that the inferred floating potential decreases significantly at 10:15, as the satellite crosses the terminator. On NorSat‐1, the negative terminals of the solar cells are grounded to the spacecraft bus while the positive side is facing the ambient plasma (Ivarsen et al., 2019). A likely explanation for the potential drop is that the solar cells facing the ambient plasma get charged positively and suddenly start collecting more electrons upon exiting solar eclipse. This would agree with findings reported by Ivarsen et al. (2019).

### 4.3 Consistency Check

**Figure 10:**
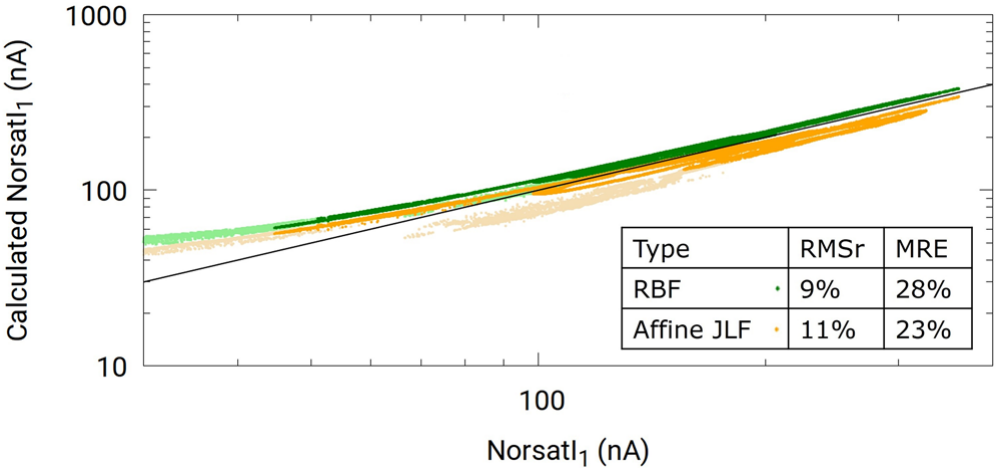
Results from the consistency check performed with in situ data following the same procedure as with the synthetic data set. Both models 1 and 2 are trained with our synthetic data, and applied to currents from the +10 V probe on NorSat‐1. Darker colors refer to inferences made with currents above 10 nA, while lighter colors refer to inferences obtained with currents between 1 and 10 nA.

In the absence of accurate and validated inferred densities and satellite potentials from NorSat‐1 data, it is not possible to confidently ascertain to what extent the inferences presented above are accurate. As an alternative, we proceed with a consistency check, following the same procedure as presented in Section 3.2 with synthetic data, but using measured currents as input. This is done by first applying models *M*1(*n*_*e*_) and *M*1(*V*_*f*_) trained with synthetic data, to infer floating potentials and densities from measured currents. Then *M*2 (also trained with synthetic data) is used to infer currents from the *M*1—inferred floating potentials and densities. If the models constructed from the synthetic data also apply to NorSat‐1 data, the inferred currents should closely reproduce the measured NorSat‐1 currents. A correlation plot of inferred against measured currents is shown in Figure 10 for the +10 V probe. In this plot, the orange and green curves show back‐inferred currents obtained with the RBF *M*2 model. For the orange curve (Affine JLF), the density used as input in *M*2 is obtained with the affine transformed JLF method. For the green curve (RBF), the density used as input in *M*2 is obtained with RBF density, while in both cases, the floating potentials are obtained with the *M*1(*V*_*f*_) model using RBF regression. The parts in lighter colors are obtained using data with a 1 nA filter, whereas the darker color parts are obtained using data with currents above 10 nA. While the graph only shows currents above 30 nA, the 1 nA filter curve extends to the left down to about 5 nA, however, these calculated +10 V probe currents plateau in this range and are far from the measured currents. This behavior is likely due to noise levels of about 10 nA, thus extra caution should be taken when using model inferences for data below 10 nA. The RMSr calculated for the 10 nA NorSat‐1 current using direct RBF density as *M*1(*n*_*e*_) is 9%, and the MRE is 28%, whereas these numbers for the affine transformed JLF densities are 11% and 23%, respectively. The calculated +10 V probe currents based on RBF regression and affine transformed JLF method nicely follow the measured +10 V probe current except for a small increase in the variance at lower currents, thus indicating that our model constructed with synthetic data set should be applicable to in situ data.

## 5 Conclusions

Two new approaches are presented and assessed, to infer plasma and satellite parameters from currents measured with multiple fixed bias needle Langmuir probes. In the first approach, inferences are made with two multivariate regression techniques, consisting of radial basis functions, and neural networks. The second approach relies on a simple affine transformation combined with a technique first proposed by Jacobsen to infer the plasma density. Yet another approach, proposed by Barjatya et al. is considered, which consists of performing nonlinear fits of measured currents, to an analytic expression involving the density, the floating potential, and the exponent *β* as fitting parameters, while the electron temperature is estimated by other means. In all cases, the accuracy of inferences is assessed on the basis of synthetic data obtained from kinetic simulations made for space‐plasma conditions representative of those encountered along the NorSat‐1 satellite. In addition to assessments based on synthetic data, a consistency check is presented, whereby densities and satellite potentials inferred from collected currents, are used as input in an inverse regression model to infer currents for one of the probes. The advantage of this consistency check is that it is applicable to both synthetic, and in situ measured currents, and in the latter case, it does not rely on a priori given inferred densities and satellite potentials. Inference consistency checks are made with both synthetic and in situ measured currents, showing excellent agreement.

The density inference methods considered in this study yield excellent results when applied to the synthetic data set. The models constructed with synthetic data are then applied to currents measured by the four m‐NLP on NorSat‐1. The density inferences from all methods show good agreement, confirming that either method should be a significant improvement over the commonly used OML approach based on *β* = 0.5. From our findings, direct RBF and the combination of Jacobsen's linear fit with *β* = 0.5 with an affine transformation, appear as being the most promising, and deserving of further study. These two methods provide inferences that are consistent and quantitatively similar, while being relatively simple and numerically efficient. The former yields the lowest maximum relative error when assessed with synthetic data, whereas the latter is the simplest method and produces inferences with comparable accuracy. The spacecraft floating potential is also inferred using RBF regression, an adapted OML approach, and the Barjatya nonlinear fit method. The adapted OML inferences are inconsistent with the measurements from NorSat‐1 data since it indicates that the satellite potential is below −6 V, while measurements indicate that the +6 V probe is collecting electron current. Conversely, spacecraft potentials inferred with RBF regression are consistent with measured currents from the +6 V biased probe, showing that the satellite potential must have been at or above −6 V for most of the one‐and‐a‐half orbital periods considered. This failure to produce acceptable values of the satellite potential using Equation 14, and the fact that the Barjatya nonlinear fit approach with *n*_*e*_, *V*_*f*_, and *β* as fitting parameters, results in *β* values appreciably larger than one, shows that in situ measurements on NorSat‐1 generally do not closely follow the empirical expression in Equation 1. One possible cause of this is that there might be an offset for the +8 V probe current which is often lower than expected for a downward concavity in *I* as a function of *V*_*b*_. Thus a re‐calibration of the instrument, if it were possible, might improve the situation.

The analysis presented here has been focused on fixed bias multi‐needle Langmuir probes, with the same dimensions as the ones mounted on NorSat‐1, to which it has been applied as a case study. We stress, however, that the simulation‐regression approach to infer space plasma parameters, is not limited to fixed bias probes or to this particular configuration of probes. With kinetic solutions capable of reproducing analytic results under conditions when they are valid, and also capable of accounting for more physics, and more realistic geometries than theories, solution libraries, training, and validation sets can just as well be constructed for different probes, mounted on satellites, operated in fixed or sweep bias voltage mode. By following standard machine learning procedures, whereby models are trained on a subset of a solution library of known independent and dependent variables, and tested by applying them to distinct subsets, we can estimate uncertainty margins specifically associated with different inference techniques. Another important strength of the proposed simulation‐regression approach is that it enables relatively straightforward incremental improvements to a model, by accounting for more physical processes or more detailed geometries; something that would be very difficult to do in a theory. Implementation of regression models and affine transformation of the Jacobsen linear fit model involves simple arithmetic expressions with pre‐calculated coefficients and can easily be programmed for onboard processing of low‐level data. These approaches, however, would require the creation of custom data sets, when applied to a given mission, so as to account for the geometry relevant to the measuring instruments, and the space environment conditions expected along a satellite orbit. In cases where probe characteristics are well approximated with analytic expressions such as Equation 1, the BNLF technique should prove fast and convenient, as it does not require extensive simulations. Custom simulation‐regression models, on the other hand, would require more computational resources, which would necessitate optimization in order to be implemented onboard a satellite. Despite their complexity, however, such models would have the advantage of being more general than models based on fits made with empirical analytic expressions. The work presented here is by no means final. The development of improved inference approaches based on simulations and regression techniques will require significantly more efforts, involving collaborations between experimentalists and modelers; an effort well worth doing, considering the cost and years of preparation involved in scientific space missions, and the possible scientific payoff.

## Data Availability

Simulation data can be accessed through https://doi.org/10.5281/zenodo.7508167.
